# Association of Genetic Polymorphisms on *VEGFA* and *VEGFR2* With Risk of Coronary Heart Disease

**DOI:** 10.1097/MD.0000000000003413

**Published:** 2016-05-13

**Authors:** Doxing Liu, Jiantao Song, Xianfei Ji, Zunqi Liu, Mulin Cong, Bo Hu

**Affiliations:** From the Emergency Department, Shandong Provincial Hospital Affiliated to Shandong University, Jinan, Shandong, China.

## Abstract

Coronary heart disease (CHD) is a cardiovascular disease which is contributed by abnormal neovascularization. *VEGFA* (*vascular endothelial growth factor A*) and *VEGFR2* (*vascular endothelial growth factor receptor 2*) have been revealed to be involved in the pathological angiogenesis. This study was intended to confirm whether single nucleotide polymorphisms (SNPs) of *VEGFA* and *VEGFR2* were associated with CHD in a Chinese population, considering pathological features and living habits of CHD patients.

Peripheral blood samples were collected from 810 CHD patients and 805 healthy individuals. Six tag SNPs within *VEGFA* and *VEGFR2* were obtained from HapMap Database. Genotyping of SNPs was performed using SNapShot method (Applied Biosystems, Foster, CA). Odd ratios (ORs) and their 95% confidence intervals (95% CIs) were calculated to evaluate the association between SNPs and CHD risk.

Under the allelic model, 6 SNPs of *VEGFA* and *VEGFR2* were remarkably associated with the susceptibility to CHD. Genotype CT of rs3025039, TT of rs2305948, and AA of rs1873077 were associated with a reduced risk of CHD when smoking, alcohol intake and diabetes were considered, while homozygote GG of rs1570360 might elevate the susceptibility to CHD (all *P* < 0.05) for patients who were addicted to smoking or those with hypertension. All of the combined effects of rs699947 (CC/CA) and rs2305948 (TT), rs3025039 (TT) and rs2305948 (TT), rs3025039 (CT) and rs1870377 (AA) had positive effects on the risk of CHD, respectively (all *P* < 0.05). By contrast, the synthetic effects of rs69947 (CA/AA) and rs1870377 (TA), rs699947 (CA) and rs7667298 (GG), rs699947 (AA) and rs7667298 (GG), rs1570360 (GG) and rs2305948 (TT), as well as rs1570360 (GG) and rs1870377 (AA) all exhibited adverse effects on the risk of CHD, respectively (all *P* < 0.05).

Six polymorphisms in *VEGFA* and *VEGFR2* may have substantial influence on the susceptibility to CHD in a Han Chinese population. Prospective cohort studies should be further designed to confirm the above conclusions.

## INTRODUCTION

Coronary heart disease (CHD) is a cardiovascular disease with high morbidity and mortality, contributing to a total of 379,559 deaths in America in 2010.^1–3^ Moreover, about 75% deaths resulted from CHD occurred in underdeveloped and developing countries.^4–6^ The development of CHD could be attributed to genetic predisposition and nongenetic risk factors, including smoking status, alcohol consumption, stress, diabetes, and lack of exercise.^7^ The interaction between genetic and nongenetic risk factors may have significant impact on the development of CHD^8–11^ and many single nucleotide polymorphisms (SNPs) that are associated with CHD have been identified by genome-wide association studies (GWAS).^12–19^

Angiogenesis dysfunction was hitherto considered as a contributor to CHD since it is related with an elevated susceptibility to atherosclerosis, hypertension and diabetes, which are the three crucial CHD-causing maladies.^20,21^ Accordingly, mutations of maladies-related SNPs within certain genes would probably render subjects more vulnerable to CHD, such as rs10491334 in CaMK4, PI (A1/A2) polymorphisms in glycoprotein IIIa and so on.^22–24^ It has been suggested that both *vascular endothelial growth factor A* (*VEGFA*) and *vascular endothelial growth factor receptor 2 (VEGFR2)* were involved in neovascularization, vasopermeability regulation, and formation of blood vessel networks.^25–29^ The 2 heredity genes also seemed to alter the risk of hypertension among targeted populations.^30–32^

*VEGFA* is located on chromosome 6 and it could express different isoforms of proteins.^33^ The *VEGF* family contains *VEGF-A*, *VEGF-B*, *VEGF-C*, *VEGF-D*, *VEGF-E*, *VEGF-F*, and placental growth factor (*PIGF*), in which *VEGF-A* is often referred as *VEGF.* Several SNPs of *VEGF-A* identified by single-stranded conformational polymorphism analysis and sequencing have been linked with the development of coronary artery disease, endometriosis, peripheral artery disorder, and lung cancer.^34–37^ Furthermore, mutations of *VEGFR2* SNPs (e.g., rs2071559) have also been demonstrated to be related with numerous diseases including tumors, rheumatoid arthritis, proliferative retinopathies, and CHD.^38–40^ However, there are few studies which are able to clarify the intrinsic relationship between *VEGFA/VEGFR2* genetic polymorphisms and the susceptibility to CHD.^41^

Bioinformatics enabled us to discover that rs7667298 is located in the promoter region of *VEGFR2* and exonic polymorphisms of rs2305948 and rs1870377 are both situated in the ligand binding region of *VEGFR2*. Meanwhile, both rs699947 and rs1570360 are located in the promoter region of *VEGFA*, while rs3025039 has been suggested to be associated with the development of CHD by formal studies. Therefore, the association between 6 *VEGFA*/*VEGFR2* genetic polymorphisms and the risk of CHD in a Chinese Han population was clarified by our study which also adjusted for several confounding factors including smoking status, alcohol consumption, hypertension and diabetes. The additive effects of the SNPs on the susceptibility to CHD were also evaluated in the 810 CHD cases and 805 healthy controls, providing us with applicable strategies for treatment of CHD.

## MATERIALS AND METHODS

### SNPs Selection

In the present study, the SNPs were obtained from unrelated Chinese population in Shanghai using the public database (HapMap). Tag SNPs were identified using the pair-wise option of Haploview 4.2 software and an r^2^ of 0.8 was set as the threshold for the analysis. Finally, 6 SNPs in *VEGFA* (rs699947, rs3025039, rs1570360) and *VEGFR2* (rs2305948, rs1870377, rs7667298) were selected. Relevant information about SNPs in *VEGFR2* and *VEGFA* is shown in Table 1.

**TABLE 1:**
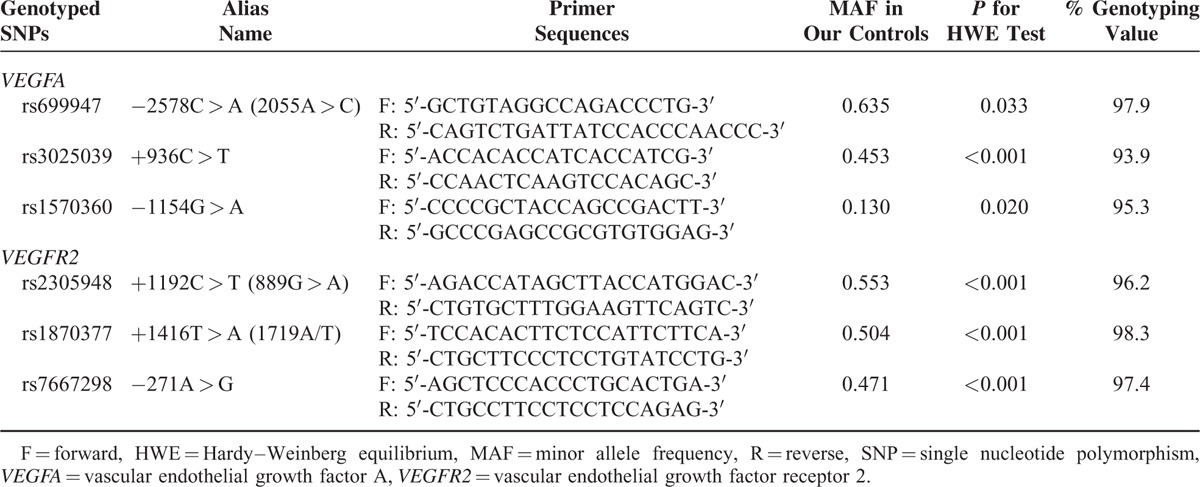
Primers of *VEGFA* and *VEGFR2* Genetic Polymorphisms for PCR Amplification

### Study Subjects

The study protocol was complied with the ethical principles of medical research for human subjects set by the Helsinki Declaration. This study was approved by both of the Review Board and the Ethics Committee of Provincial Hospital Affiliated to Shandong University (Jinan, China).^42^ This study incorporated 810 unrelated CHD patients (577 males and 233 females) and 805 unrelated healthy volunteers (586 males and 219 females). Patients were consecutively recruited from the Provincial Hospital Affiliated to Shandong University (Jinan, China) between July 2012 and September 2014. All patients were guaranteed as nonsyndromic to avoid interferences of other diseases on the study results. All participants were diagnosed using ultrasonography and confirmed by surgery. CHD patients were incorporated if they satisfied the following requirements: coronary angiography revealed that more than 50% of stenosis was present in at least 1 of 3 main blood arteries (right coronary artery, left circumflex artery, and left anterior descending coronary artery) and stenosis can be identified in 2 left main coronary arteries; patients suffered from syndromes of unstable angina pectoris: angina was present when at rest and lasted for more than 20 minutes, or newly developed (<2 months) severe angina, or aggravation angina with increased intensity, duration, and frequency; clinical syndromes of myocardial infarction appeared, such as consistent and intense chest pain for more than 30 minutes, characteristic change of electrocardiograph (e.g., ST segment of 2–3 adjacent leads elevated or depressed for ≥1 mm, and left bundle branch block emerged) and abnormal rise of myocardial enzymology. Healthy controls were included when they were diagnosed by coronary angiography to be without CHD. Besides, all the participants, including CHD patients and healthy controls, were all excluded if they regularly take statins or other lipid lowering drugs within 2 months; were operated with percutaneous coronary intervention (PCI) for less than 6 months when plasma specimens were gathered; were confirmed to carry severe cardiovascular diseases, including congestive heart failure (CHF), valvular heart disease, cardiomyopathy, and malignant arrhythmia; had hepatic and kidney function obstacles, connective tissue disease, tumors, and so on. The control group was matched with the case group with respect to age and sex and all subjects were recruited from the same hospital during the same period. All participants in the study have signed the informed consents and they were selected from Han Chinese. Detailed clinical data of all patients were collected using a standard data collection form.

### Testing CHD Indexes

A complete set of vascular risk factors obtained from the subjects are recorded in Table 2, including body mass index (BMI), smoking status, alcohol consumption, hypertension and diabetes, total cholesterol (TC), triglyceride (TG), high-density lipoprotein cholesterol (HDC-L), and low-density lipoprotein cholesterol (LDC-L). Arterial hypertension was recorded if the average systolic pressures of 3 independent blood pressure tests were more than 140 mm Hg or the average diastolic pressure was higher than 90 mm Hg. Diabetes mellitus (DM) was suggested as a fasting glucose of 7.8 or 11.1 mmol/L 2 hours after oral glucose challenge. Furthermore, subjects were defined to have smoking history if they continuously smoked for more than 2 years. Subjects who consumed >2 ounce of liquor or >4 ounce of beer per day were considered to have alcoholic consumption history. Subjects and their parents were personally questioned by trained interviewers using a structured questionnaire to obtain information about maternal DM and hypertension status.

**TABLE 2:**
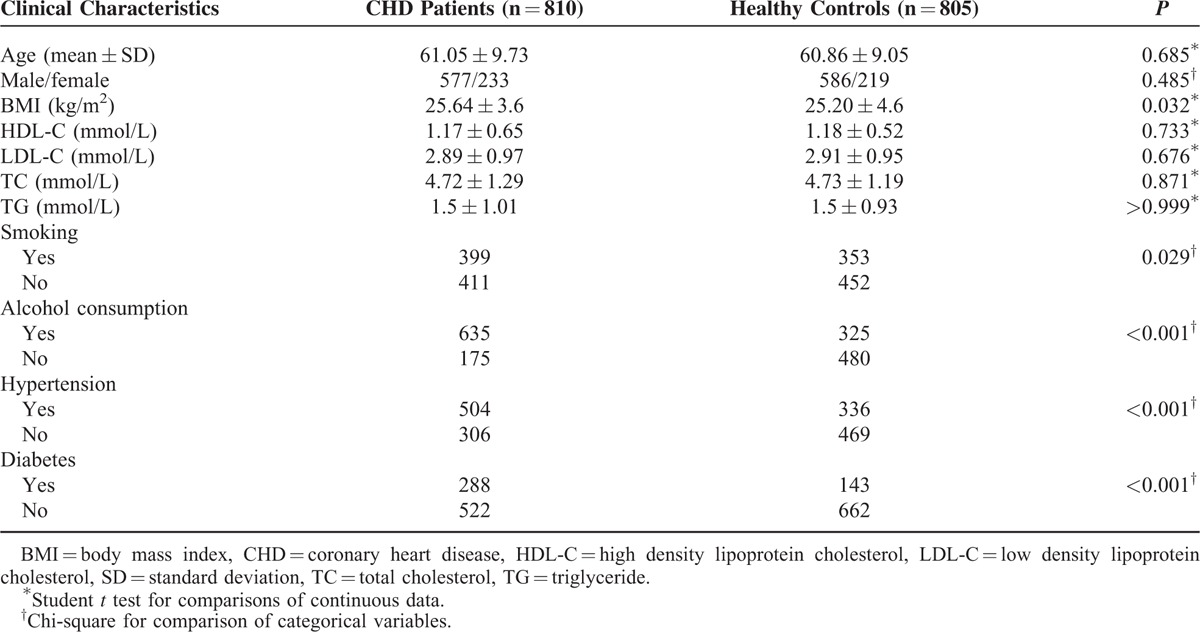
Comparisons of Selective Characteristics Between CHD Patients and Healthy Controls

### Sample Collection

Peripheral venous blood samples (10 mL) were collected from all patients using vacutainer tubes in the morning. Blood samples (5 mL) for genetic analyses were transferred into tubes which contained ethylenediamine tetra-acetic acid (EDTA). Genomic DNAs were isolated using genomic DNA extraction kit (QIA amp DNA Blood Mini Kit, Qiagen, Berlin, Germany) under the manufacturer's instructions.

### Genetic Analysis

Five milliliters of venous blood samples were stored in a sterile tube with heparin sodium inside. The mixtures were subsequently centrifuged at 3000 rpm for 15 minutes at room temperature in order to get the separated plasma samples, which were then immediately stored at −20°C. Subsequently, genomic DNAs were extracted from frozen peripheral blood samples via a QIAmp Blood Mini Kit (Qiagen, Inc., Valencia, CA) according to the manufacturer's protocols. Tag SNPs were amplified with 9700 polymerase chain reaction (PCR) System (Applied Biosystems) with the primers designed by Primer 3 software (Table 1). The PCR reaction mixture (10 μL) included 1 μL genomic DNA, 0.6 μL 5 μM designed primers, 0.4 μL 10 mM dNTP mix, 0.1 μL Taq DNA polymerase, 1 μL Taq DNA polymerase buffer, and 6.3 μL ultrapure water. The reaction mixture of *VEGFA* was initially desaturated at 94°C for 4 minutes; desaturated at 94°C (30 seconds), annealed at 62°C (30 seconds), and extended at 72°C (45 seconds) for 35 cycles; terminated at 72°C for 2 minutes and finally reserved at 4°C. Similarly, the reaction mixture of *VEGFR2* was predesaturated at 95°C for 5 minutes; then desaturated at 94°C (30 seconds), annealed at 61°C (30 seconds), and extended at 72°C (30 seconds) for 35 cycles; terminated at 72°C for 10 minutes and finally reserved at 4°C. The resultant PCR products were purified by the addition of 1.7 U shrimp alkaline phosphatase (SAP). Finally, the SnapShot assay (Applied Biosystems) was performed to confirm the genotypes of all DNA samples.

### Statistical Analysis

Continuous variables were expressed in the form of mean ± standard deviation (SD) whereas categorical variables were expressed as frequencies and percentages. Comparisons of continuous variables between the case and control group were carried out using the Student *t* test. The Chi-square (*χ*^2^ test) was used to analyze distribution differences in gender, smoking status and alcohol consumption status between the case and control group. Apart from that, the (*χ*^2^ test was applied to verify whether genotype frequencies were complied with Hardy–Weinberg equilibrium (HWE). Deviations from HWE were also estimated using the *χ*^2^ test for each SNP. Besides that, 3 genetic models, namely, the allelic (M vs W), dominant (MW/MM vs WW), and recessive (MM vs WW/MW) models, were applied to evaluate the association between *VEGFA*/*VEGFR2* genetic polymorphisms and CHD susceptibility using odd ratios (ORs) and their 95% confidence intervals (CIs). ORs along with their corresponding 95% CIs calculated by the *χ*^2^ test were also used to ascertain the correlation between polymorphisms and risk of CHD after adjust for confounding factors including age, gender, BMI, smoking status, hypertension, diabetes, and drinking status. All the statistical analyses were conducted by SPSS18.0 statistical software (SPSS, Inc, Chicago, IL) and a 2-sided *P*-value of less than 0.05 was considered as statistically significant.

## RESULTS

### Characteristics of the Study Population

A total of 810 CHD patients (male/female: 2.46) averaging (61.05 ± 9.73) years old and 805 healthy volunteers (male/female: 2.67) with averaging (60.86 ± 9.05) years old were included in our study. Compared with the control group, significantly more CHD patients were accompanied with smoking habits (49% vs 43.9%) and alcoholic consumption (78.4% vs 40.4%) as well as complications of diabetes (35.6% vs 17.8%) and hypertension (62.2% vs 41.7%) (all *P* < 0.05) (Table 2). However, no significant intergroup difference was observed between the case and control group in HDL, VDL, TG, and TC levels (*P* > 0.05).

### Associations of *VEGFA* Genetic Polymorphisms With Risk of CHD

Genotype distributions of the 3 SNPs located in *VEGFA* among the case and control group are summarized in Table 3. All loci in the case and the control group were complied with HWE (*P* > 0.05). Under the allelic model, T allele of rs3025039, A allele of rs1570360, and C allele of rs699947 were all significantly associated with higher CHD risk (T vs C, OR = 1.97, 95% CI: 1.64–2.37; G vs A, OR = 0.51, 95% CI: 0.44–0.58; A vs C, OR = 0.78, 95% CI: 0.67–0.89). As suggested by the dominant model, genotype TT/CT of rs3025039 also conferred an enhancive risk of CHD (TT/CT vs CC, OR = 2.35, 95% CI: 1.90–2.91), while genotypes of rs1570360 and rs699947 were associated with a lower risk of CHD (GG/AG vs AA, OR = 0.39, 95% CI: 0.32–0.48; AA/CA vs CC, OR = 0.75, 95% CI: 0.58–0.98).

**TABLE 3:**
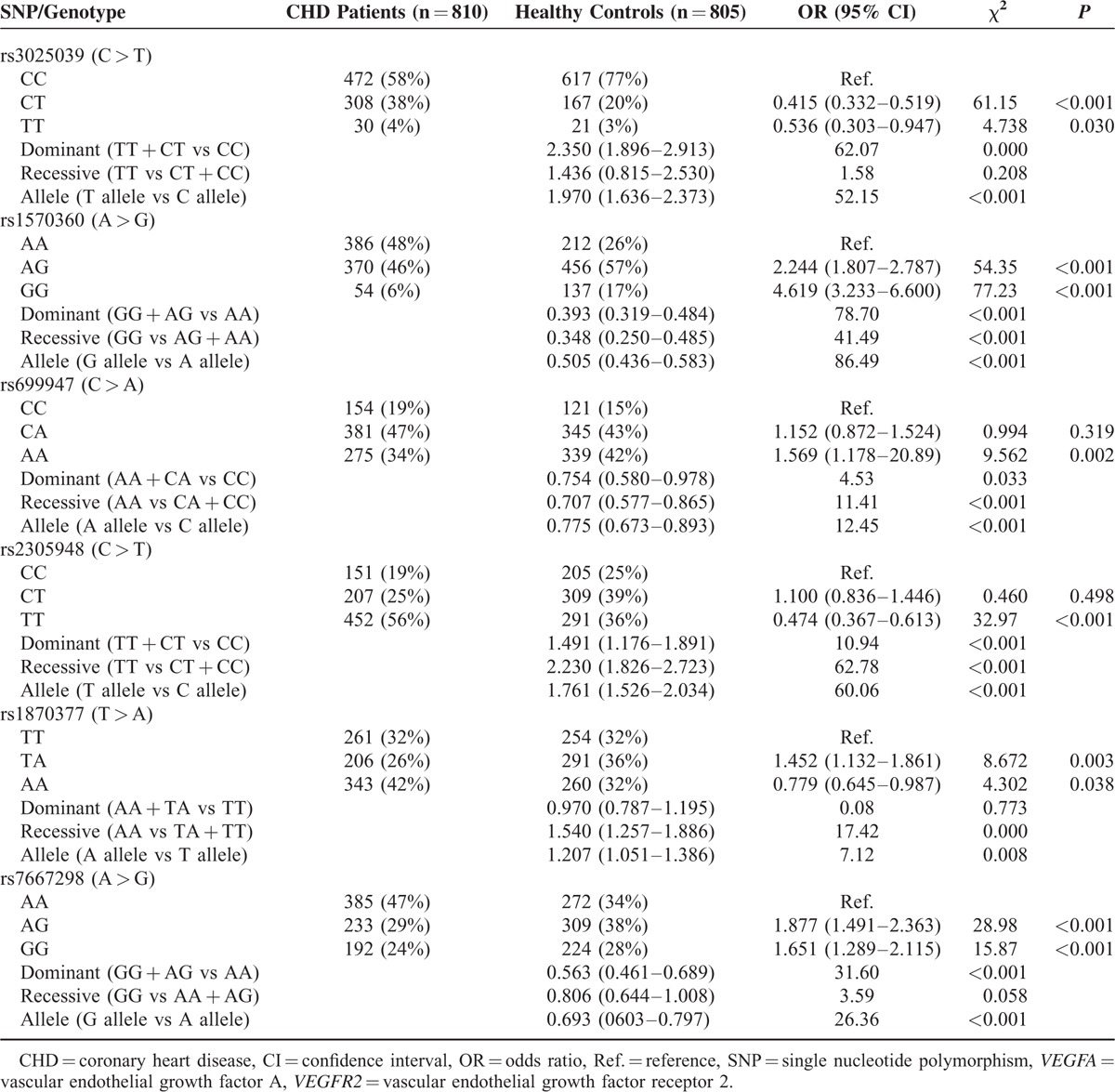
Associations Between Six Polymorphisms of *VEGFA* and *VEGFR2* and Risk of CHD

### Associations of *VEGFR2* Genetic Polymorphisms With Risk of CHD

The polymorphism of *VEGFR2* rs2305948 (C > T) exhibited significant correlations with higher risk of CHD (TT/CT vs CC., OR = 1.49, 95% CI: 1.18–1.89, *P* < 0.001; T vs C, OR = 1.76, 95% CI: 1.52–2.03, *P* < 0.001, respectively) (Table 3). The allelic variant of rs1870377 (T > A) appeared to dramatically increase CHD risk (A vs T, OR = 1.21, 95% CI: 1.05–1.39, *P* = 0.008) as well. By contrast, individuals with mutations of rs7667298 (A > G) might have significantly reduced risk of CHD (GG/AG vs AA: OR = 0.56, 95% CI = 0.46–0.70, *P* < 0.001; G vs A: OR = 0.69, 95% CI = 0.60–0.80, *P* < 0.001).

### Correlation Between *VEGFA*/*VEGFR2* Genotypes and CHD Risk Among Populations Stratified by Smoking Status

For both smokers and nonsmokers, subjects with genotype AA of rs699947 were associated with an increased risk of CHD when compared with those carrying CC genotype (smoker, OR = 1.72, 95% CI: 1.12–2.65; nonsmoker, OR = 1.45, 95% CI: 1.01–2.18) (Table 4). Identical trends were observed for homozygote GG and heterozygote AG of rs1570360 irrespective of smoking status (all *P* < 0.05). Conversely, either smokers or nonsmokers with genotypes (CT vs CC; TT vs CC) of rs3025039 and rs2305948 exhibited significant associations with a reduced susceptibility to CHD (all OR < 1; *P* < 0.05). As for rs1870377, heterozygote TA conferred elevated risk of CHD among nonsmokers, whereas homozygote AA served as a protective factor for CHD (TA vs TT, OR = 1.44, 95% CI: 1.03–2.01; AA vs TT, OR = 0.63, 95% CI: 0.45–0.87). Dissimilarly, both homozygote GG and heterozygote AG were correlated with higher CHD risk, among smoking and nonsmoking populations (GG vs AA, OR = 1.53, 95% CI: 1.06–2.20; AG vs AA, OR = 1.88, 95% CI: 1.37–2.59).

**TABLE 4:**
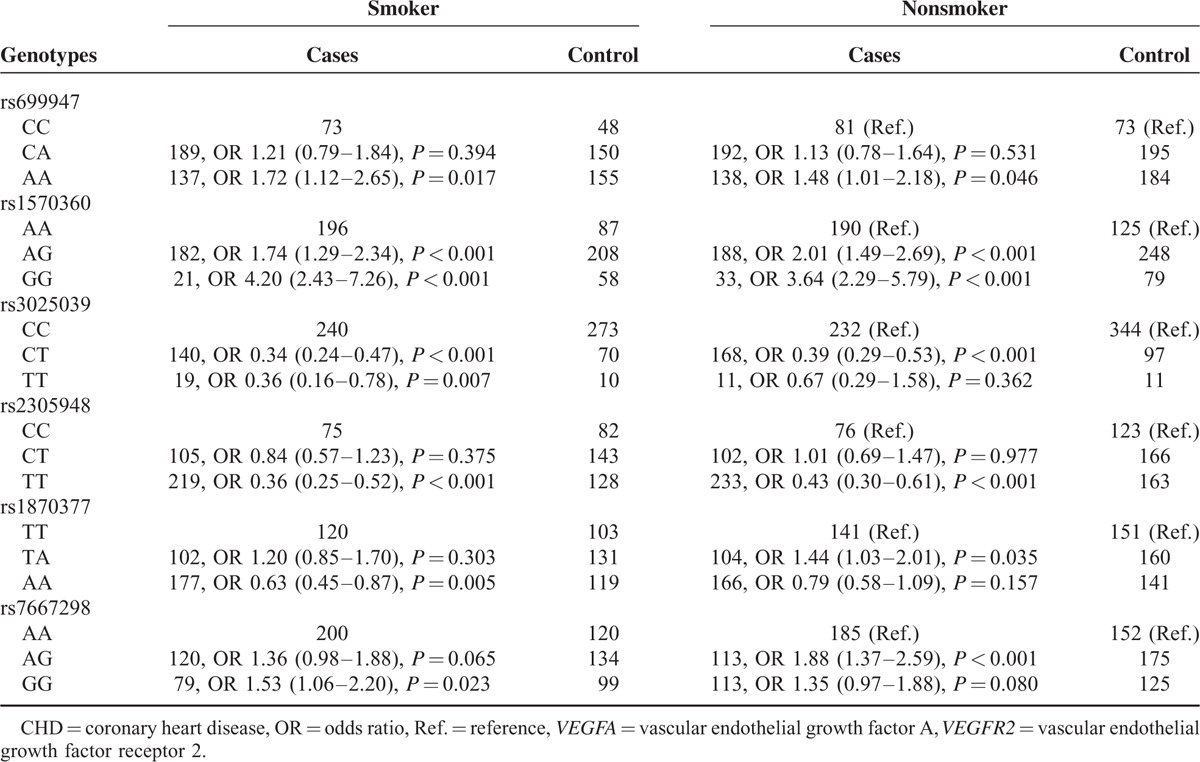
Correlation Between *VEGFA*/*VEGFR2* Genotypes and Smoking Among CHD Patients and Healthy Controls

### Correlation Between *VEGFA*/*VEGFR2* Genotypes and CHD Risk Among Populations Stratified by Alcoholic Consumption

For individuals with alcohol consumptions (Table 5), genotypes of rs699947 (CA vs CC; AA vs CC), rs1570360 (AG vs AA), rs3025039 (CT vs CC; TT vs CC), rs2305948 (CT vs CC; TT vs CC), rs1870377 (TA vs TT; AA vs TT), and rs7667298 (AG vs AA; GG vs AA) were all correlated with a lower susceptibility to CHD (OR = 0.25, 95% CI: 0.16–0.40; OR = 0.33, 95% CI: 0.20–0.52; OR = 0.40, 95% CI: 0.29–0.56; OR = 0.07, 95% CI: 0.05–0.09; OR = 0.14, 95% CI: 0.06–0.30; OR = 0.18, 95% CI: 0.11–0.28; OR = 0.10, 95% CI: 0.06–0.14; OR = 0.23, 95% CI: 0.16–0.35; OR = 0.14, 95% CI: 0.09–0.21; OR = 0.19, 95% CI: 0.14–0.26; OR = 0.25, 95% CI: 0.11–0.56). Among individuals without alcohol consumptions (Table 5), homozygotes of rs699947 (AA vs CC) and rs1570360 (GG vs AA) showed positive effects on the development of CHD (OR = 2.17, 95% CI: 1.30–3.62; OR = 3.13, 95% CI: 1.73–5.65). Nonetheless, homozygotes of rs3025039 (TT vs CC), rs2305948 (TT vs CC), rs1870377 (AA vs TT), and rs7667298 (GG vs AA) were all associated with a decreased risk of CHD (OR = 0.25, 95% CI: 0.10–0.62; OR = 0.38, 95% CI: 0.24–0.60; OR = 0.63, 95% CI: 0.41–0.95; OR = 0.42, 95% CI: 0.18–0.97).

**TABLE 5:**
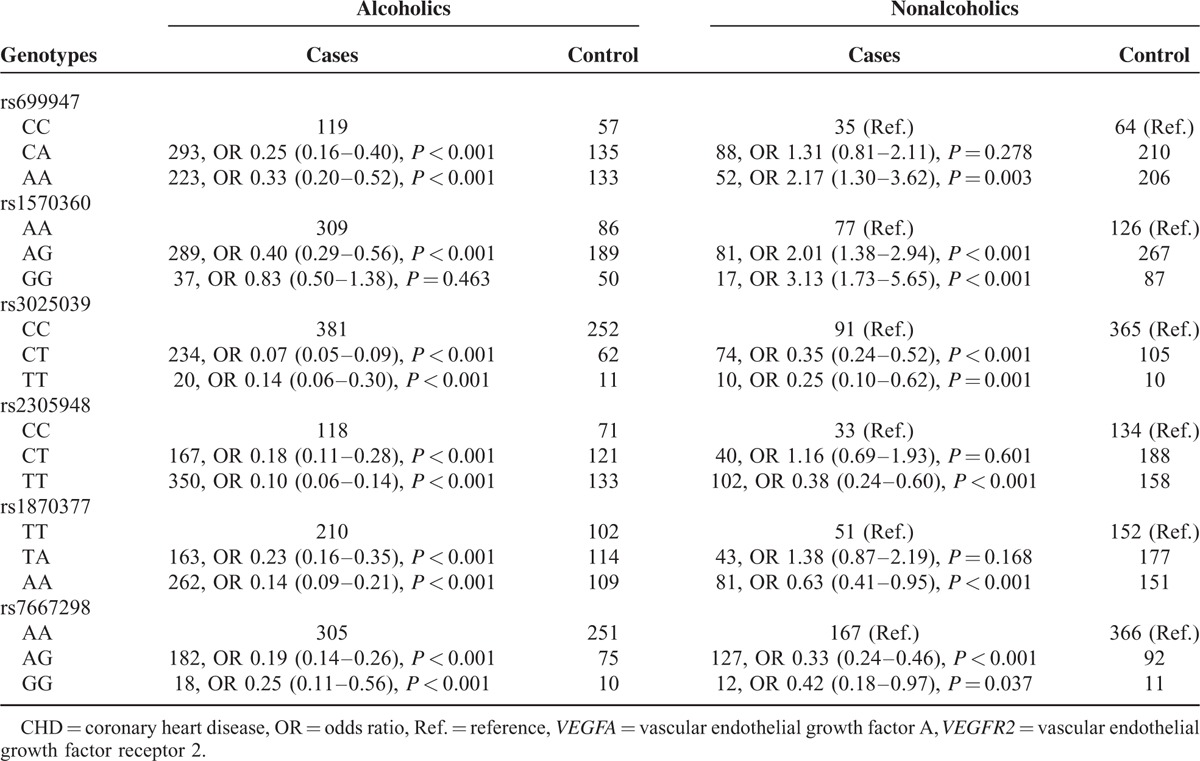
Correlation Between *VEGFA*/*VEGFR2* Genotypes and Alcohol Intake Among CHD Patients and Healthy Controls

### Correlation Between *VEGFA*/*VEGFR2* Genotypes and CHD Risk Among Populations Stratified by Diabetic Status

Diabetic participants with either homozygotes or heterozygotes of rs699947, rs3025039, rs2305948, rs1870377, and rs7667298 tended to be less susceptible to CHD than those with homozygotes CC, CC, CC, TT, and AA, respectively (all *P* < 0.05) (Table 6). Nondiabetic participants with genotypes of rs699947 (AA vs CC) and rs1570360 (AG vs AA; GG vs AA) appeared to be more readily subjected to CHD (OR = 2.79, 95% CI: 1.87–4.16; OR = 2.22, 95% CI: 1.73–2.85; OR = 4.73, 95% CI: 3.08–7.26). However, genotypes of the other 4 SNPs were still linked with lessened risk of CHD among nondiabetics participants (all *P* < 0.05).

**TABLE 6:**
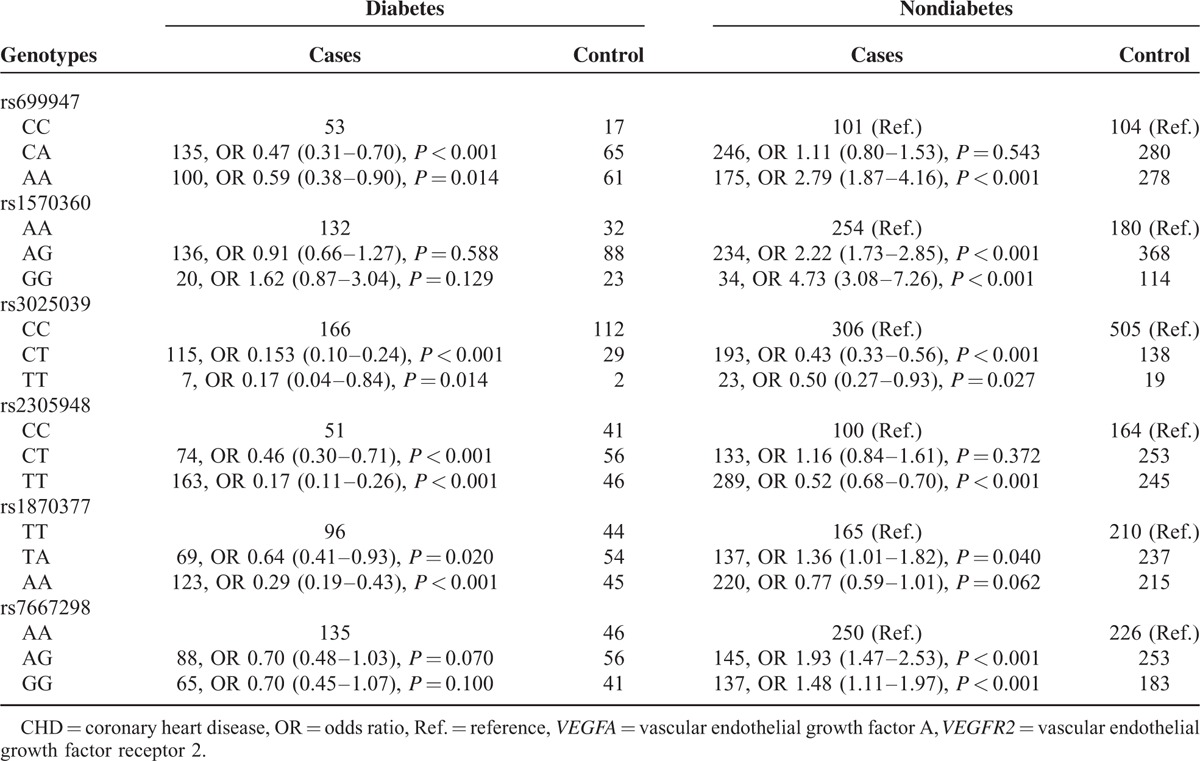
Interaction Between *VEGFA* and *VEGFR2* Genotypes With Diabetes in CHD Cases and Controls

### Correlation Between *VEGFA*/*VEGFR2* Genotypes and CHD Risk Among Populations Stratified by Hypertension

Unlike stratification by diabetics status, results from participants with hypertension (Table 7) suggested that heterozygote CT and homozygote TT of rs3025039 had opposite effects on CHD risk when compared with homozygote CC (OR = 1.90, 95% CI: 1.10–3.28; OR = 0.24, 95% CI: 0.11–0.53). Concerning rs1570360, carriers of homozygote GG were significantly associated with higher-risk of CHD than those of AA (OR = 2.02, 95% CI: 1.25–3.27). Genotypes of additional SNPs all had protective effects on CHD development among subjects with high blood pressure (all *P* < 0.05). For nonhypertensive patients, up to 5-fold increased risk of CHD was associated with GG genotype of rs1570360 (GG vs AA: OR = 5.22, 95% CI = 3.00–9.01, *P* < 0.001). Genotypes of rs699947 (AA vs CC), rs1570360 (AG vs AA), rs1870377 (TA vs TT), and rs7667298 (AG vs AA; GG vs AA) were related with about 2-fold incremental risk of CHD (OR = 1.79, 95% CI: 1.16–2.76; OR = 2.48, 95% CI: 1.81–3.40; OR = 1.61, 95% CI: 1.11–2.35; OR = 1.80, 95% CI: 1.28–2.52; OR = 1.75, 95% CI: 1.21–2.53). On the contrary, genotypes (TT vs CC) of the remaining SNPs (i.e., rs3025039 and rs2305948) were associated with a reduced CHD risk (OR = 0.42, 95% CI: 0.18–0.97; OR = 0.39, 95% CI: 0.26–0.57).

**TABLE 7:**
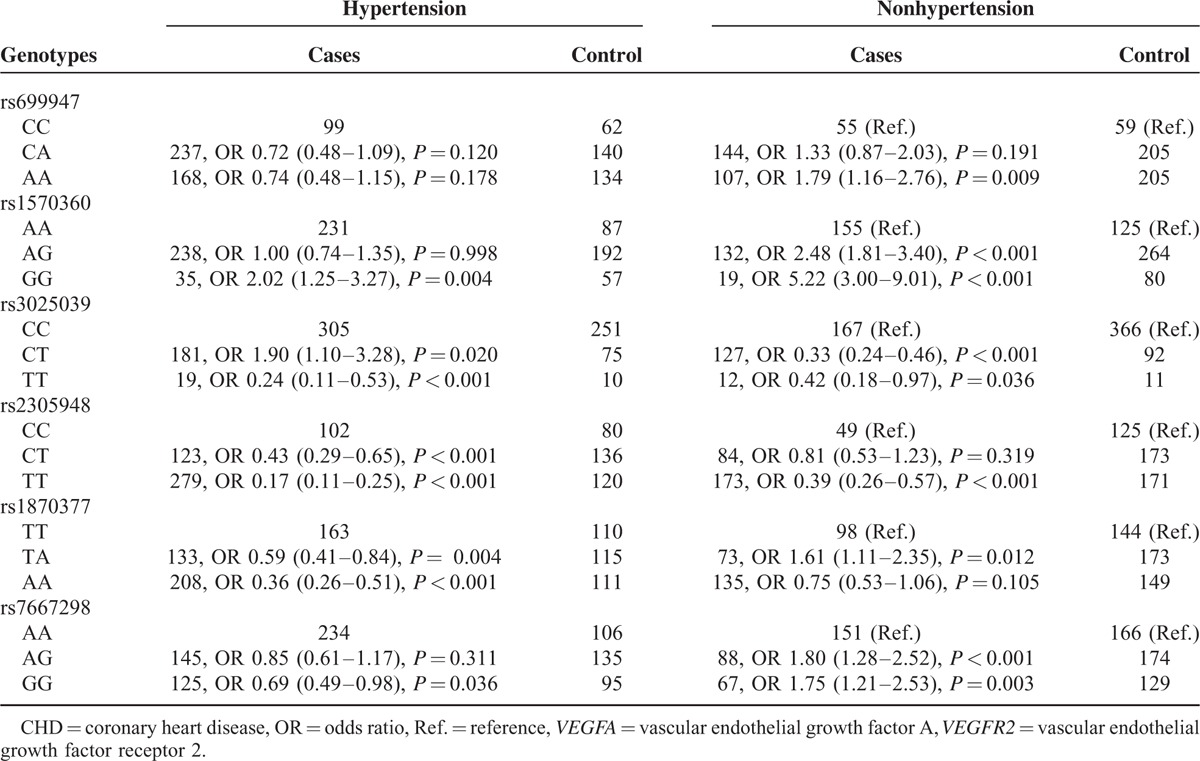
Interaction Between *VEGFA* and *VEGFR2* Genotypes With Hypertension in CHD Cases and Controls

### Association of Combined Genotypes of *VEGFA* (rs699947) and *VEGFR2* (rs2305948, rs1873077, and rs7667298) With Risk of CHD

The combined *VEGFA* (rs699947 CC/CA) and *VEGFR2* (rs2305948 TT) genotype both presented protective effects on developing CHD (CCCT vs CCCC: OR = 0.51, 95% CI = 0.28–0.95, *P* < 0.05; CATT vs CCCC: OR = 0.38, 95% CI = 0.22–0.67, *P* < 0.001) (Table 8). On the contrary, coactions of *VEGFA* (rs699947 CA/AA) and *VEGFR2* (rs1870377 TA) were associated with higher risk of CHD (CATA vs CCTT: OR = 1.71, 95% CI = 1.04–2.81, *P* = 0.034; AATA vs CCTT: OR = 2.01, 95% CI = 1.20–3.38, *P* = 0.008). However, subjects with CA of rs699947 together with TT of rs1870377 might be less liable to CHD (CATT vs CCTT: OR = 0.41, 95% CI = 0.24–0.72, *P* = 0.002). Furthermore, subjects with 6 combined genotypes of *VEGFA* (rs699947) and *VEGFR2* (rs7667298) including CCAG, CCGG, CAGG, AAAA, AAAG, and AAGG were more susceptible to CHD in comparison to CCAA (all *P* < 0.05).

**TABLE 8:**
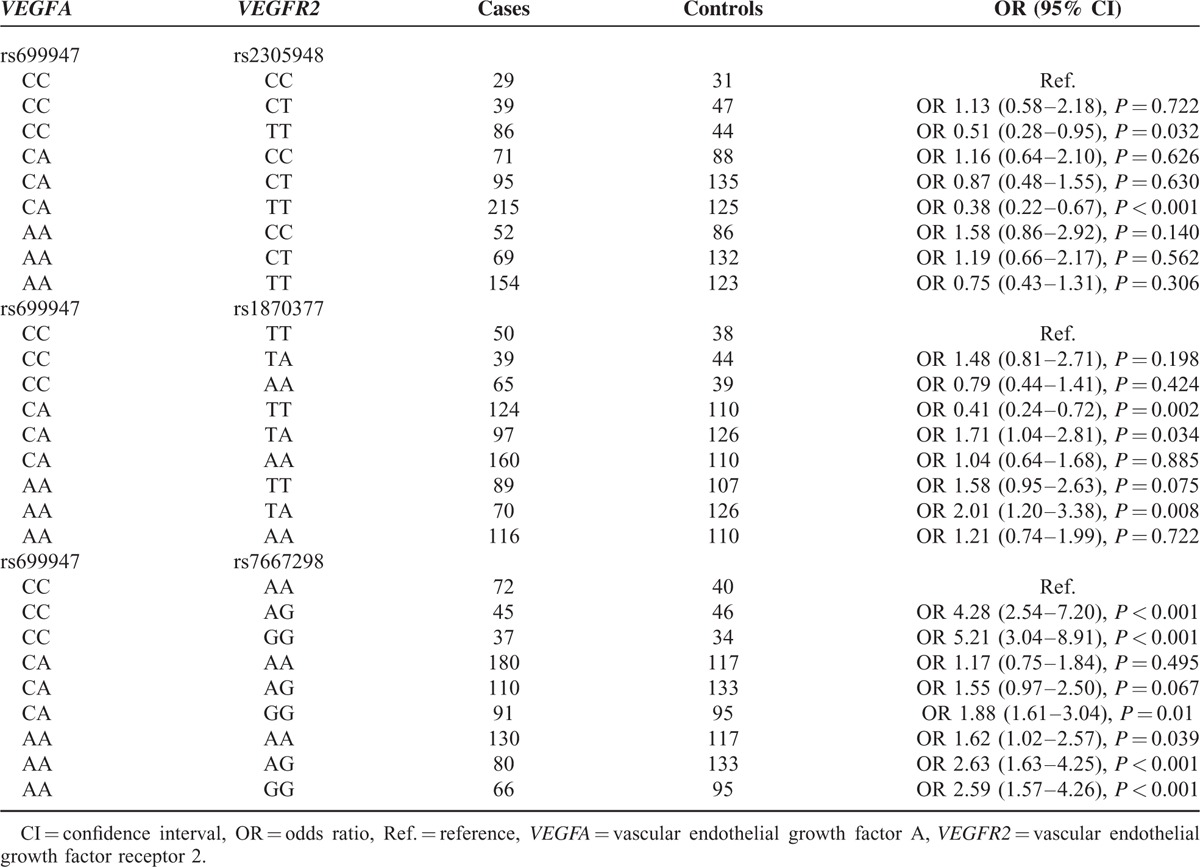
The Combined Effects of Genetic Polymorphisms Within *VEGFA* (rs699947) and *VEGFR2* (rs2305948, rs1870377, and rs7667298) on Risk of Coronary Heart Disease

#### Association of Combined Genotypes of *VEGFA* (rs1570360) and *VEGFR2* (rs2305948, rs1873077, and rs7667298) With Risk of CHD

When compared with AACC carriers, carriers of *VEGFA* (rs1570360 AA) and *VEGFR2* (rs2305948 TT) were more likely to develop CHD, while carriers of AGCC, AGCT, GGCC, GGCT, and GGTT appeared to be less vulnerable to CHD (all *P* < 0.05). Almost all potential combined genotypes of *VEGFA* (rs1570360) and *VEGFR2* (rs1870377), except for AAAA were positively associated with CHD susceptibility compared with AATT (all OR > 1, *P* < 0.05). Similarly, 8 combined genotypes of *VEGFA* (rs1570360) and *VEGFR2* (rs7667298), except for AAAG, were also linked with higher incidence of CHD (Table 9).

**TABLE 9:**
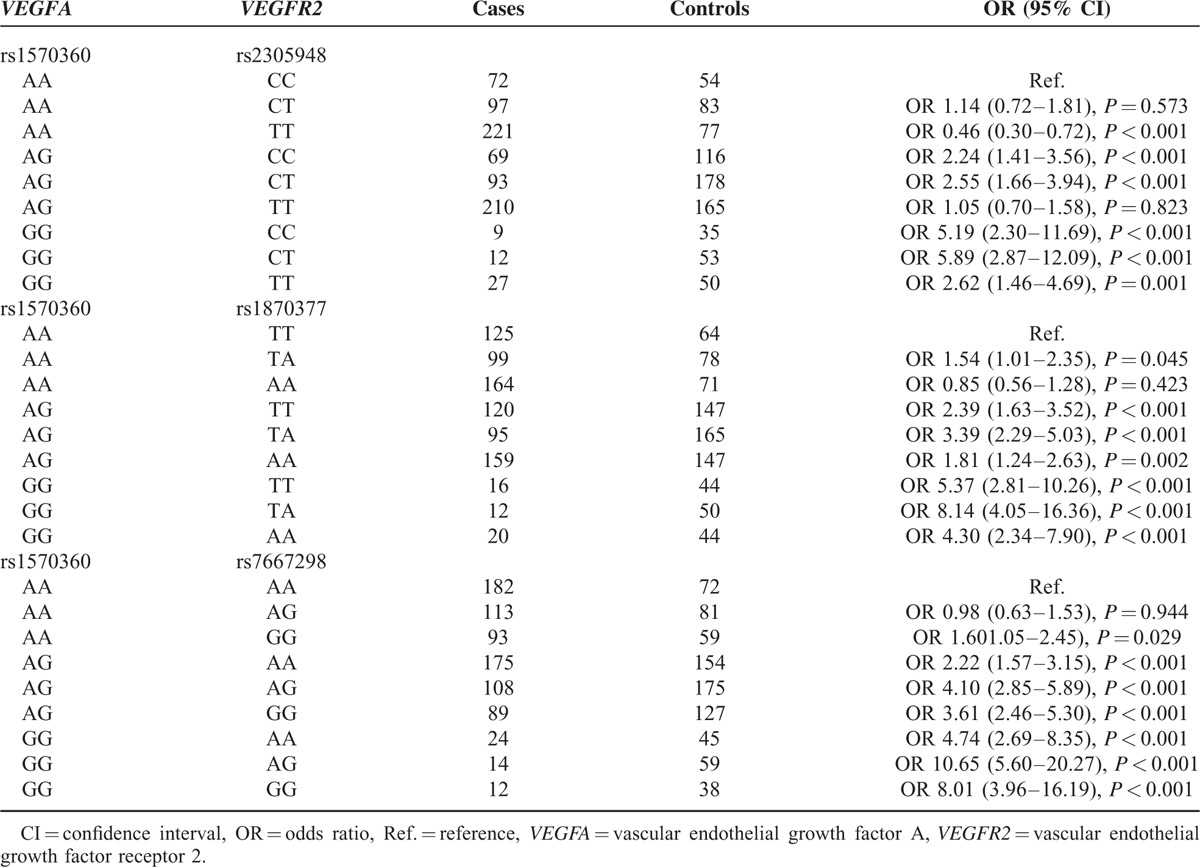
The Combined Effect of Genetic Polymorphisms Within *VEGFA* (rs1570360) and *VEGFR2* (rs2305948, rs1870377, and rs7667298) on Risk of Coronary Heart Disease

### Association of Combined Genotypes of *VEGFA* (rs3025039) and *VEGFR2* (rs2305948, rs1873077, and rs7667298) With Risk of CHD

The combination of *VEGFA* rs3025039 (CC/CT) and *VEGFR2* (rs2305948 CC/CT/TT) genotypes were associated with a decreased risk of CHD (*P* < 0.05). Besides, *VEGFA* (rs3025039 CT) and *VEGFR2* (rs1870377 TT/TA/AA) exhibited the same reduced risk with OR less than 1, despite that the opposite tendency was exhibited by CCTA when compared with CCTT (*P* < 0.05). Remarkable associations were also observed between *VEGFA* (rs3025039 CC), *VEGFR2* (rs7667298 AG/GG) genotype as well as CTAA and risk of CHD with CCAA as the control (all *P* < 0.05) (Table 10).

**TABLE 10:**
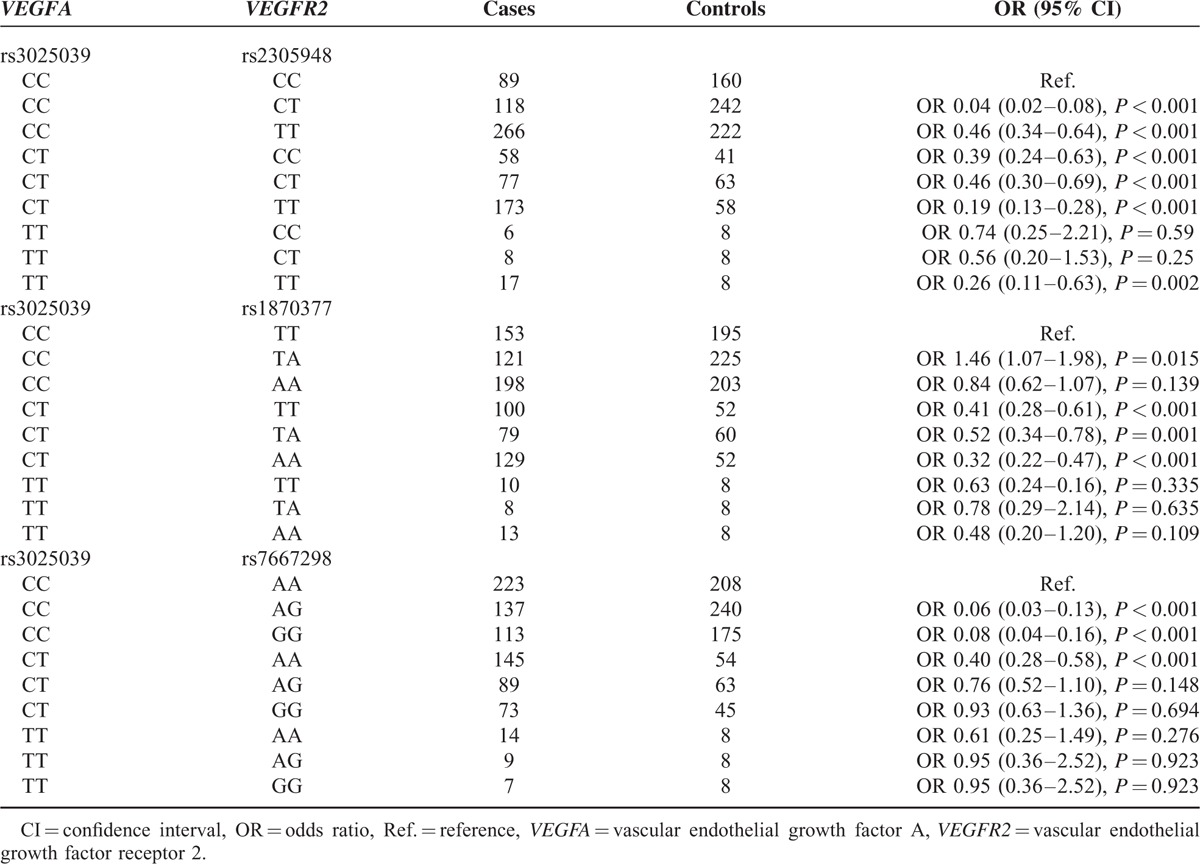
The Combined Effects of Genetic Polymorphisms Within *VEGFA* (rs3025039) and *VEGFR2* (rs2305948, rs1870377, and rs7667298) on Risk of Coronary Heart Disease

## DISCUSSION

CHD is the most common type of heart disease which is mainly caused by narrow coronary artery and inadequate blood supply. A large number of studies have suggested that the mechanism of CHD is linked with familial heredity. However, the etiological factors for CHD are still vague. VEGFA has been illustrated to promote the differentiation, proliferation, and migration of microvascular endothelial cells by binding to their receptors, thus facilitating microvessel formation and development.^43^ Moreover, VEGF could not only promote the progress of blood vessel recanalization and the establishment of collateral circulation, but also enhance the dependent vasodilatation of the endothelial cells all of which are closely linked with CHD. In addition, it is reported that VEGFA might play a vital role during the process of epithelial–mesenchymal transition (EMT) and regulate the formation of endocardial cushion.^44^ Since VEGF is involved in many physiological activities, temporal and spatial expressions of VEGF should be precisely regulated to prevent abnormal VEGF expressions which could trigger cardiovascular disorder including CHD. The above hypothesis has been confirmed by animal models which suggested that appropriate timing and dosage of VEGF during heart development was of vital importance in order to avoid cardiovascular defects.^45,46^

Recently, several studies have investigated the relationship between *VEGFA* gene polymorphisms and CHD risk.^41,44,47^ A previous case–control study conducted by Wang et al^44^ found that 2 VEGF SNPs (rs699947 and rs3025039) were not associated with CHD in a Chinese population. On the contrary, a study by Han et al^41^ indicated that *VEGFA* rs3025039 polymorphisms were significantly related with CHD risk among another Chinese population. Furthermore, a meta-analysis conducted by Griffin et al^47^ indicated that *VEGFA* polymorphisms may not be associated with CHD. To further ascertain the association between SNPs within *VEGFA* and CHD, we genotyped three tag SNPs (rs699947, rs3025039, and rs1570360) in this study and discovered that all the 3 SNPs were remarkably correlated with the susceptibility to CHD.

Previous studies also have demonstrated that VEGFR2 which is a kind of tyrosine kinase receptor is essential for hematopoiesis and angiogenesis since it regulates the mitogenic and chemotactic roles of VEGFA in endothelial cells.^48^ Subsequently, the combination of VEGFA and VEGFR2 is able to activate downstream signaling pathways (e.g., MAPK, Akt, and eNOS), which are essential for stimulating angiogenesis.^40^ Furthermore, intralesion angiogenesis was associated with atherosclerotic plaques that could contribute to acute coronary syndromes.^40,49^ Hence, endothelial dysfunction and abnormal angiogenesis resulted from VEGFA and VEGFR2 may increase the risk of CHD. As suggested by Wang et al, *VEGFR2* polymorphisms may act as genetic markers for identifying CHD risk in a Chinese population,^40^ indicating that 2 SNPs of *VEGFR2* could significantly affect the binding efficiency of *VEGF* to *VEGFR2*. Nonetheless, another research revealed no significant association between *VEGFR2* and CHD in a Japanese population with Kawasaki disease.^50^ In this study, we selected 3 SNPs of *VEGFR2* to investigate the relation between *VEGFR2* and CHD in a Chinese population, suggesting that *VEGFR2* polymorphisms (rs2305948, rs1870377, and rs7667298) were associated with CHD risk. Inconsistency between the 2 studies may arise from differences in genetic backgrounds, disease mechanisms, sample size, and other confounding factors.^51^

Of note, our results provided a comprehensive analysis of *VEGFA* and *VEGFR2* genetic polymorphisms in both CHD and non-CHD patients. Samples in this study were obtained from individuals with multiple cardiovascular risk factors because only few patients undergoing bypass surgery were found to have only one isolated risk factor. Therefore, a regression analysis was firstly conducted to confirm that SNPs of *VEGFA* or *VEGFR2* were still associated with the risk of CHD when the effects of smoking, alcoholic consumption, diabetes, or hypertension were excluded. The above risk factors could somewhat aggravate the incidence of CHD with the presence of hereditary factors. Furthermore, after excluding effects imposed by SNPs of *VEGFA* or *VEGFR2*, mutations of SNPs within other genes could still elevate subjects’ susceptibility to CHD.

Despite the above strengths, several limitations should be addressed with regard to this study. First of all, the retrospective nature of this study was featured by exploration of an established fact based on preexisting recordings, rather than a designed or randomized experiment beforehand (e.g., prospective studies). This characteristic may appear as a source of bias (e.g., information bias and selection bias) in that recalled baseline information (e.g., smoking severity and alcoholic consumption) might be affected by confounding factors, such as the participants’ subjectivity during the process of data collection. Moreover, our sample size should be enlarged to further elucidate the role of *VEGFA* and *VEGFR2* genetic polymorphisms in CHD risk. It was because that the distinctions of genetic factors and environmental backgrounds actually existed between the study group and Chinese populations of a larger size or other ethnic groups. Thus, the investigation results might not be applicable to other Chinese populations or additional ethnic groups. Besides, it could not be ignored that the uniqueness of samples might exaggerate the correlation between the studied 6 genetic polymorphisms and CHD risk, or neglect the role of some other vital polymorphisms within *VEGFA* and *VEGFR2* in susceptibility to CHD. In addition, this study has not addressed other potential etiologies of CHD, especially for some other genes that are associated with *VEGFA* and *VEGFR2* (e.g., *CD14*). Therefore, further researches, including large prospective cohort studies or combined meta-analyses, should be designed to address these limitations.

## CONCLUSION

In summary, the present study indicated that polymorphisms in *VEGFA* [rs3025039 (C > T), rs1570360 (A > G), and rs699947 (C > A)] and *VEGFR2* [rs2305948 (C > T), rs1870377 (T > A), and rs7667298 (A > G)] were notably correlated with altered CHD susceptibility in the Han Chinese population, when potential effects of living habits (e.g., smoking and alcohol intake) or complications (e.g., hypertension and diabetes) were removed. Therefore, mutations of the SNPs could be applied clinically as genetic markers. Besides, harmful living habits could aggravate CHD development and relevant complications could also be CHD-causing parameters.

## References

[1] Huang CJ Hsieh MH Hou WH Depression, antidepressants, and the risk of coronary heart disease: a population-based cohort study. *Int J Cardiol* 2013; 168:4711–4716. 23948112 10.1016/j.ijcard.2013.07.173

[2] Yu Q Shao H He P World scientific collaboration in coronary heart disease research. *Int J Cardiol* 2013; 167:631–639. 23068572 10.1016/j.ijcard.2012.09.134

[3] Williams RA Cardiovascular disease in African American women: a health care disparities issue. *J Natl Med Assoc* 2009; 101:536–540. 19585921 10.1016/s0027-9684(15)30938-x

[4] Mozaffarian D Benjamin EJ Go AS Heart disease and stroke statistics—2015 update: a report from the American Heart Association. *Circulation* 2015; 131:e29–e322. 25520374 10.1161/CIR.0000000000000152

[5] Gaziano TA Bitton A Anand S Growing epidemic of coronary heart disease in low- and middle-income countries. *Curr Probl Cardiol* 2010; 35:72–115. 20109979 10.1016/j.cpcardiol.2009.10.002 PMC2864143

[6] Go AS Mozaffarian D Roger VL Heart disease and stroke statistics—2014 update: a report from the American Heart Association. *Circulation* 2014; 129:e28–e292. 24352519 10.1161/01.cir.0000441139.02102.80 PMC5408159

[7] Hrira MY Chkioua L Slimani A Hsp70-2 gene polymorphism: susceptibility implication in Tunisian patients with coronary artery disease. *Diagn Pathol* 2012; 7:88–92. 22834788 10.1186/1746-1596-7-88 PMC3558340

[8] Wang Q Molecular genetics of coronary artery disease. *Curr Opin Cardiol* 2005; 20:182–188. 15861005 10.1097/01.hco.0000160373.77190.f1 PMC1579824

[9] Kitsios G Zintzaras E ACE (I/D) polymorphism and response to treatment in coronary artery disease: a comprehensive database and meta-analysis involving study quality evaluation. *BMC Med Genet* 2009; 10:50–65. 19497121 10.1186/1471-2350-10-50 PMC2700093

[10] Kangas-Kontio T Huotari A Ruotsalainen H Genetic and environmental determinants of total and high-molecular weight adiponectin in families with low HDL-cholesterol and early onset coronary heart disease. *Atherosclerosis* 2010; 210:479–485. 20056223 10.1016/j.atherosclerosis.2009.12.022

[11] Arnett DK Baird AE Barkley RA Relevance of genetics and genomics for prevention and treatment of cardiovascular disease: a scientific statement from the American Heart Association Council on Epidemiology and Prevention, the Stroke Council, and the Functional Genomics and Translational Biology Interdisciplinary Working Group. *Circulation* 2007; 115:2878–2901. 17515457 10.1161/CIRCULATIONAHA.107.183679

[12] Kondo H Ninomiya T Hata J Angiotensin I-converting enzyme gene polymorphism enhances the effect of hypercholesterolemia on the risk of coronary heart disease in a general Japanese population: the Hisayama study. *J Atheroscler Thromb* 2015; 22:390–403. 25342476 10.5551/jat.24166

[13] Song C Liu B Yang D Association between interleukin-6 gene −572G>C polymorphism and coronary heart disease. *Cell Biochem Biophys* 2015; 71:359–365. 25312476 10.1007/s12013-014-0206-z

[14] Huang Y Ye H Hong Q Association of CDKN2BAS polymorphism rs4977574 with coronary heart disease: a case-control study and a meta-analysis. *Int J Mol Sci* 2014; 15:17478–17492. 25268619 10.3390/ijms151017478 PMC4227174

[15] Li Z Jun Y Zhong-Bao R Association between MTHFR C677T polymorphism and congenital heart disease. A family-based meta-analysis. *Herz* 2015; 40 (Suppl. 2):160–167. 25256053 10.1007/s00059-014-4144-8

[16] Zain M Awan FR Cooper JA Association of TLL1 gene polymorphism (rs1503298, T > C) with coronary heart disease in PREDICT, UDACS and ED cohorts. *J Coll Physicians Surg Pak* 2014; 24:615–619. 25233961

[17] Luo JY Ma YT Xie X Association of intercellular adhesion molecule1 gene polymorphism with coronary heart disease. *Mol Med Rep* 2014; 10:1343–1348. 24993975 10.3892/mmr.2014.2360

[18] Fan SL Li X Chen SJ ABCA1 rs4149313 polymorphism and susceptibility to coronary heart disease: a meta-analysis. *Ann Hum Genet* 2014; 78:264–276. 24942079 10.1111/ahg.12068

[19] Zhou K Wang Y Peng W Genetic variants of the endothelial NO synthase gene (eNOS) may confer increased risk of sporadic congenital heart disease. *Genet Mol Res* 2014; 13:3805–3811. 24938467 10.4238/2014.May.16.4

[20] Santulli G Angiopoietin-like proteins: a comprehensive look. *Front Endocrinol (Lausanne)* 2014; 5:4–9. 24478758 10.3389/fendo.2014.00004 PMC3899539

[21] D’Alessio A Moccia F Li JH Angiogenesis and vasculogenesis in health and disease. *Biomed Res Int* 2015; 2015:126582. 26171386 10.1155/2015/126582 PMC4478295

[22] Santulli G Cipolletta E Sorriento D CaMK4 gene deletion induces hypertension. *J Am Heart Assoc* 2012; 1:e001081. 23130158 10.1161/JAHA.112.001081 PMC3487344

[23] Lanni F Santulli G Izzo R The Pl(A1/A2) polymorphism of glycoprotein IIIa and cerebrovascular events in hypertension: increased risk of ischemic stroke in high-risk patients. *J Hypertens* 2007; 25:551–556. 17278970 10.1097/HJH.0b013e328013cd67

[24] Galasso G Santulli G Piscione F The GPIIIA PlA2 polymorphism is associated with an increased risk of cardiovascular adverse events. *BMC Cardiovasc Disord* 2010; 10:41–47. 20846430 10.1186/1471-2261-10-41 PMC2954874

[25] Moradzadegan A Vaisi-Raygani A Nikzamir A Angiotensin converting enzyme insertion/deletion (I/D) (rs4646994) and Vegf polymorphism (+405G/C; rs2010963) in type II diabetic patients: association with the risk of coronary artery disease. *J Renin Angiotensin Aldosterone Syst* 2015; 16:672–680. 24505095 10.1177/1470320313497819

[26] Cai J Ahmad S Jiang WG Activation of vascular endothelial growth factor receptor-1 sustains angiogenesis and Bcl-2 expression via the phosphatidylinositol 3-kinase pathway in endothelial cells. *Diabetes* 2003; 52:2959–2968. 14633857 10.2337/diabetes.52.12.2959

[27] Holmes K Roberts OL Thomas AM Vascular endothelial growth factor receptor-2: structure, function, intracellular signalling and therapeutic inhibition. *Cell Signal* 2007; 19:2003–2012. 17658244 10.1016/j.cellsig.2007.05.013

[28] Stuttfeld E Ballmer-Hofer K Structure and function of VEGF receptors. *IUBMB Life* 2009; 61:915–922. 19658168 10.1002/iub.234

[29] Matsumoto T Mugishima H Signal transduction via vascular endothelial growth factor (VEGF) receptors and their roles in atherogenesis. *J Atheroscler Thromb* 2006; 13:130–135. 16835467 10.5551/jat.13.130

[30] Palmirotta R Ferroni P Ludovici G VEGF-A gene promoter polymorphisms and microvascular complications in patients with essential hypertension. *Clin Biochem* 2010; 43:1090–1095. 20621078 10.1016/j.clinbiochem.2010.06.020

[31] Kim JJ Vaziri SA Rini BI Association of VEGF and VEGFR2 single nucleotide polymorphisms with hypertension and clinical outcome in metastatic clear cell renal cell carcinoma patients treated with sunitinib. *Cancer* 2012; 118:1946–1954. 21882181 10.1002/cncr.26491 PMC4124607

[32] Jain L Sissung TM Danesi R Hypertension and hand-foot skin reactions related to VEGFR2 genotype and improved clinical outcome following bevacizumab and sorafenib. *J Exp Clin Cancer Res* 2010; 29:95–102. 20630084 10.1186/1756-9966-29-95 PMC2913951

[33] Shahin RM Abdelhakim MA Owid ME A study of VEGF gene polymorphism in Egyptian patients with diabetic retinopathy. *Ophthalmic Genet* 2015; 1–6. 24502825 10.3109/13816810.2014.881508

[34] Biselli PM Guerzoni AR de Godoy MF Vascular endothelial growth factor genetic variability and coronary artery disease in Brazilian population. *Heart Vessels* 2008; 23:371–375. 19037583 10.1007/s00380-008-1057-6

[35] Jiang Y Tang JY Wu Y [Vascular endothelial growth factor gene polymorphisms and the risk of endometriosis: a systematic review]. *Zhonghua Fu Chan Ke Za Zhi* 2012; 47:179–184. 22781068

[36] Bleda S De Haro J Varela C Impact of VEGF polymorphisms on the severity of peripheral artery disease in diabetic patients. *Growth Factors* 2012; 30:277–282. 22762535 10.3109/08977194.2012.703664

[37] Liu C Zhou X Gao F Correlation of genetic polymorphism of vascular endothelial growth factor gene with susceptibility to lung cancer. *Cancer Gene Ther* 2015; 22:312–316. 26065377 10.1038/cgt.2015.24

[38] Szarejko-Paradowska A Gluba-Brzozka A Pietruszynski R Assessment of the relationship between selected cardiovascular risk factors and the indices of intima-media thickness and coronary artery calcium score in various stages of chronic kidney disease. *Int Urol Nephrol* 2015; 47:2003–2012. 26494632 10.1007/s11255-015-1132-8

[39] Elshabrawy HA Chen Z Volin MV The pathogenic role of angiogenesis in rheumatoid arthritis. *Angiogenesis* 2015; 18:433–448. 26198292 10.1007/s10456-015-9477-2 PMC4879881

[40] Wang Y Zheng Y Zhang W Polymorphisms of KDR gene are associated with coronary heart disease. *J Am Coll Cardiol* 2007; 50:760–767. 17707181 10.1016/j.jacc.2007.04.074

[41] Han X Liu L Niu J Association between VEGF polymorphisms (936c/t, −460t/c and −634 g/c) with haplotypes and coronary heart disease susceptibility. *Int J Clin Exp Pathol* 2015; 8:922–927. 25755796 PMC4348824

[42] Glas J Seiderer J Bues S IRGM variants and susceptibility to inflammatory bowel disease in the German population. *PLoS One* 2013; 8:e54338. 23365659 10.1371/journal.pone.0054338 PMC3554777

[43] Tettamanti G Malagoli D Benelli R Growth factors and chemokines: a comparative functional approach between invertebrates and vertebrates. *Curr Med Chem* 2006; 13:2737–2750. 17073625 10.2174/092986706778521986

[44] Wang E Wang Z Liu S Polymorphisms of VEGF, TGFbeta1, TGFbetaR2 and conotruncal heart defects in a Chinese population. *Mol Biol Rep* 2014; 41:1763–1770. 24443223 10.1007/s11033-014-3025-9

[45] Miquerol L Langille BL Nagy A Embryonic development is disrupted by modest increases in vascular endothelial growth factor gene expression. *Development* 2000; 127:3941–3946. 10952892 10.1242/dev.127.18.3941

[46] Carmeliet P Ferreira V Breier G Abnormal blood vessel development and lethality in embryos lacking a single VEGF allele. *Nature* 1996; 380:435–439. 8602241 10.1038/380435a0

[47] Griffin HR Hall DH Topf A Genetic variation in VEGF does not contribute significantly to the risk of congenital cardiovascular malformation. *PLoS One* 2009; 4:e4978. 19308252 10.1371/journal.pone.0004978 PMC2654913

[48] Zhong S Wu J Cui Y Vascular endothelial growth factor from Trimeresurus jerdonii venom specifically binds to VEGFR-2. *Biochimie* 2015; 116:1–7. 26107411 10.1016/j.biochi.2015.06.011

[49] Tenaglia AN Peters KG Sketch MH Jr Neovascularization in atherectomy specimens from patients with unstable angina: implications for pathogenesis of unstable angina. *Am Heart J* 1998; 135:10–14. 9453515 10.1016/s0002-8703(98)70336-9

[50] Kariyazono H Ohno T Khajoee V Association of vascular endothelial growth factor (VEGF) and VEGF receptor gene polymorphisms with coronary artery lesions of Kawasaki disease. *Pediatr Res* 2004; 56:953–959. 15470196 10.1203/01.PDR.0000145280.26284.B9

[51] International HapMap Consortium. The International HapMap Project. *Nature* 2003; 426:789–796. 14685227 10.1038/nature02168

