# Association of Genetic Polymorphisms on *VEGFA* and *VEGFR2* With Risk of Coronary Heart Disease: Erratum

**DOI:** 10.1097/01.md.0000484496.43819.1a

**Published:** 2016-08-19

In the article “Association of Genetic Polymorphisms on *VEGFA* and *VEGFR2* With Risk of Coronary Heart Disease”,^1^ which appeared in Volume 95, Issue 19 of *Medicine*, Dr. Dongxing Liu's name was originally misspelled. The article has since been corrected online.
